# Detecting Excitations of Pipes, Ropes, and Bars Using Piezo Sensors and Collecting Information Remotely

**DOI:** 10.3390/s25051444

**Published:** 2025-02-27

**Authors:** Matteo Cirillo, Enzo Reali, Giuseppe Soda

**Affiliations:** 1 Dipartimento di Fisica, Università di Roma “Tor Vergata”, 00133 Roma, Italy; reali@roma2.infn.it; 2 Pipe Monitoring Corporation (PIMOC), Via Cola di Rienzo 212, 00192 Roma, Italy; pinogsoda@gmail.com

**Keywords:** piezoelectric sensors, pipes monitoring, signal handling and transmission, pulse transmission, acoustic excitations

## Abstract

An investigation of a non-invasive method to detect defects and localize excitations in metallic structures is presented. It is shown how signals generated by very sensitive piezo sensor assemblies, secured to the metallic elements, can allow for space localization of excitations and defects in the analyzed structures. The origin of the piezo excitations are acoustic modes generated by light percussive excitations whose strength is of the order of tenths of a newton and that provide piezo signal amplitudes of a few hundred millivolts. Tests of the detection scheme of the excitations are performed on steel ropes, iron pipes, and bars with lengths in the range of 1–6 m with the sensor output signal shaped in the form of a clean pulse. It is shown that the signals generated by the piezo assemblies, when adequately shaped, can feed the input of an RF transmitter, which in turn transfers information to a remote receiver whose readout allows for remotely analyzing information collected on the metallic elements. Considering the voltage amplitude of the signals (of the order of 300 mV) generated by the piezo sensors as a result of very light percussive excitations, the low power required for transmitting data, and the low cost of the sensing and transmitting assembly, it is conceivable that our devices could detect excitations generated even tens of kilometers away and allow for setting up an array of sensors for controlling in real time the status of pipe networks.

## 1. Introduction

Piezoelectric sensors and devices cover a wide range of applications in fundamental science, engineering, and technology [1,2,3] and represent a unique tool when the issue is to detect very small displacements and excitations of solid bodies [4]. Today, at least three relevant fields for possible applications of piezo devices exist, and our efforts are targeted at proposing possible solutions. Regarding the type of application, the reason why we will herein concentrate our experiments on specific shapes/geometries of the metallic elements will be clear. 

The first issue motivating our interest is monitoring the security of oil and gas pipelines; this is a problem of paramount relevance for avoiding fraud and related environmental disasters along conducts taking oil or gas from sources to refineries or from refineries to distributors [5,6,7,8,9,10,11]. Fraud is committed by producing holes in the pipelines from which the product is extracted. This criminal activity produces economic and environmental damages quantifiable in the order of several hundred million dollars per year. Setting up a reliable protocol for detecting, in real time, harmful operations on the pipelines would constitute relevant progress for the safety of oil/gas distribution. Naturally, the system to be set up for the detection must consider the fact that a pipeline’s length can be thousands of kilometers and the transmission of the signals of the sensors to adequate receivers must go through ether. At this time, a system remotely monitoring the status of pipeline networks and providing information in real time on the location/origin of the problems does not exist.

Another relevant issue concerning the localization of defects in metals and cables is the control of the status of rail tracks [12,13,14,15,16,17] and steel ropes of cable cars [18,19,20,21,22,23]. In both these cases, a sensitive and non-invasive system for localizing, well ahead of problems, the quality and the status of ropes and rail tracks would be extremely helpful and prevent problems due to cracks and defects. A terrible accident that happened in Italy on the Stresa Mottarone cable car in May 2021 has attracted much attention to the research for defects localization on the ropes. The ropes that we use are those also provided for cable car construction. The high velocities achieved by trains, however, also require paramount attention on the quality, performances, and status of conservation of the rail tracks.

Finally, the degradation of iron and steel embedded in concrete is a problem that is also gathering much attention from the structural engineering side [24,25,26,27,28]. This problem has become particularly important in Italy after the Morandi bridge disaster in Genova in August 2018. The authors of this present publication have active collaborations with highway construction companies (ANAS, TOTO Construction) in order to find possible solutions for the problem, namely checking the status of the steel embedded in concrete structures [29].

For the physical cases and necessities described above, the primary requirements in the search for detection systems are adequate sensitivity, reliability, the possibility of operating over long distances the sensor devices, and, naturally, the possibility of collecting information remotely. In this present paper, it is described how a piezoelectric assembly, requiring very limited power for its operation, could allow for detecting the defects of bars, ropes, and pipelines and also to localize the presence of external excitations along these structures. The basic principle for all cases is the detection of acoustic excitations. The signals generated by the piezo units caused by the excitations are transformed into rectangular impulses, and time delays associated with them allow for determining the presence of defects and excitations. It has also been shown how the impulses generated by our measurement protocol are transmitted through the ether and received remotely to handle the information. Evidence will be provided that our sensor devices and detection system have all the ingredients necessary for application to cases of noticeable interest.

The remainder of this paper is structured as follows. In the next section, the basic hardware and the detection principle of the sensor assembly are described and it is shown that it can detect very weak excitations when secured to steel pipes; in Section 3, it is demonstrated how the signals of the sensors can be treated to generate clean pulses, allowing for the straight detection of time delays and transmission of information at large distances. The pulses originated by the piezos as described in Section 3 are then employed to characterize pipes, bars, and ropes in Section 4. In Section 5, evidence of excitations and defect localization using our measuring protocol is provided in steel ropes and bars. In Section 6, we draw the conclusions of the work.

## 2. The Sensor Assembly

**Figure 1:**
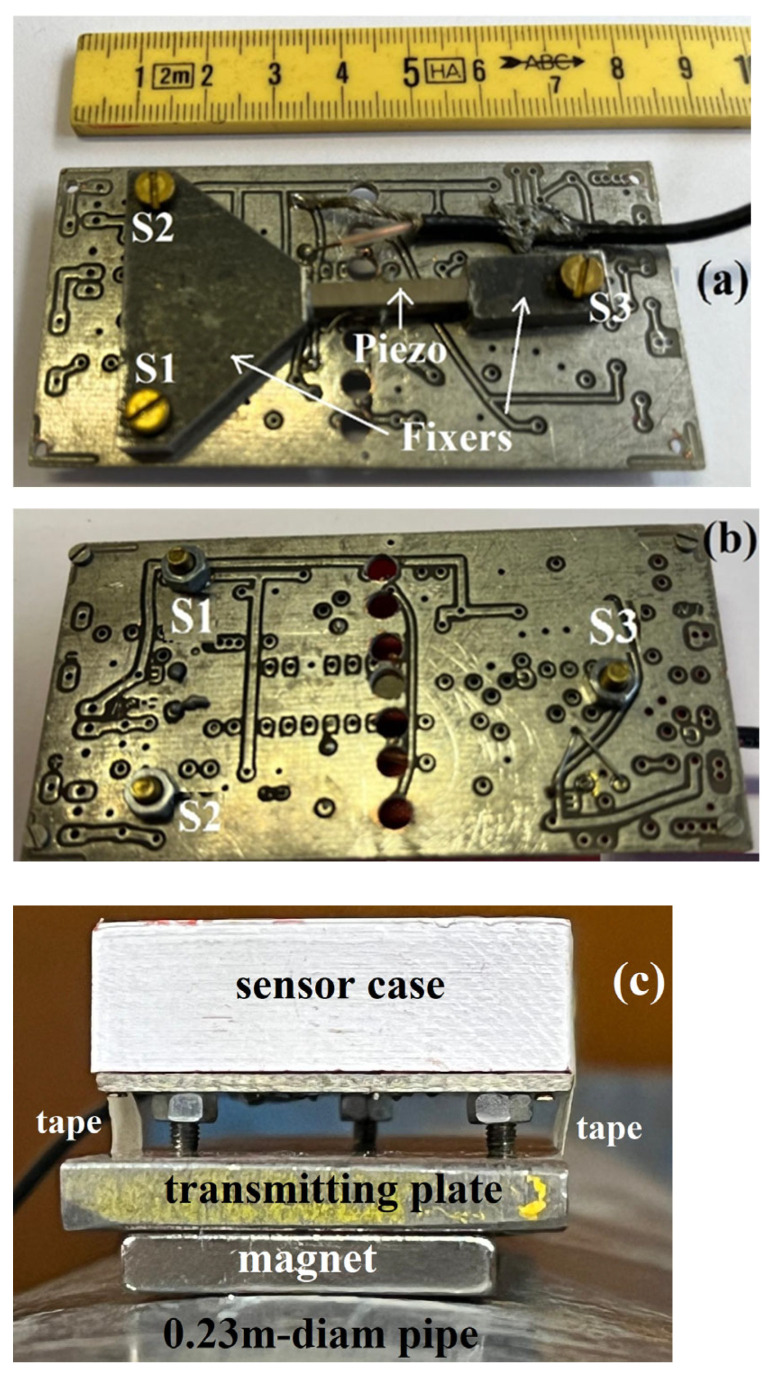
Views of our sensor assembly. (**a**) Image of the top showing the piezo element, the fixers, and the screws securing those to a PCB; (**b**) the back of the PCB showing the end of the screws; (**c**) cross-section of the sensor assembly positioned on a 0.23 m-diameter pipe—below the sensor case three fixing screws contact the “transmitting plate”. The strong magnet (B = 0.45 T) fixes the plate to the pipe and robust tape (indicated by side) secures the case to the transmitting plate.

In Figure 1a, the mounting assembly of our piezo sensor is shown. The piezo element is fixed on a Printed Circuit Board (PCB) by holding it in mechanical contact with two pieces of metal (the fixers): the piezo and its fixers are shown by arrows in the figure. A tiny amount of silver paste (Agar Quick drying g3020) is used to enhance the contact between the piezo and fixers. The piezo elements we used were part of a stock purchased from the company Riccardo Beyerle s.p.a. (Milano, Italy). The piezo material is described in ref. [30]; specifically, it is Pb(Zr_0.52_Ti_0.48_)O_3_. The role of the fixers is, other than firmly positioning the piezo on the board, to transmit the mechanical stresses to the piezo: the two metal fixers contacting the piezo are secured by three screws (S1, S2, and S3 in the photo) to the PCB and these are tightened up by a nut on the back of the board, as shown in Figure 1b. 

The assembly of Figure 1a,b was covered by a plastic case and firmly tied by robust tape on an iron flat metal plate (l = 14 cm, w = 4 cm, t = 0.4 cm) as shown in the cross section of Figure 1c where the sensor assembly is placed over a 0.23 m-diameter pipe. The three screws shown in Figure 1b as S1, S2, and S3 contact the metal plate, which we call the “transmission plate”; this plate, in turn, is secured to the metallic elements whose excitations we intend to trace by placing two strong parallelepiped-shaped magnets (l = 3 cm, w = 1 cm, t = 0.5 cm) between the transmission plate and the metallic elements. The magnets generate an induction field B = 0.45 T.

The shape of the transmitting plate will always remain flat on the side where it must contact the three screws (and through the screws, the fixers, and the sensor); however, the rest of it can be shaped for the exigence of contacting objects of different solid geometries. For the experiments that we will present here, all devoted to testing iron or iron-based metallic elements, using the strong magnets and the metal plate for securing the sensor assemblies to the elements resulted in them being mechanically stable and reliable. 

**Figure 2:**
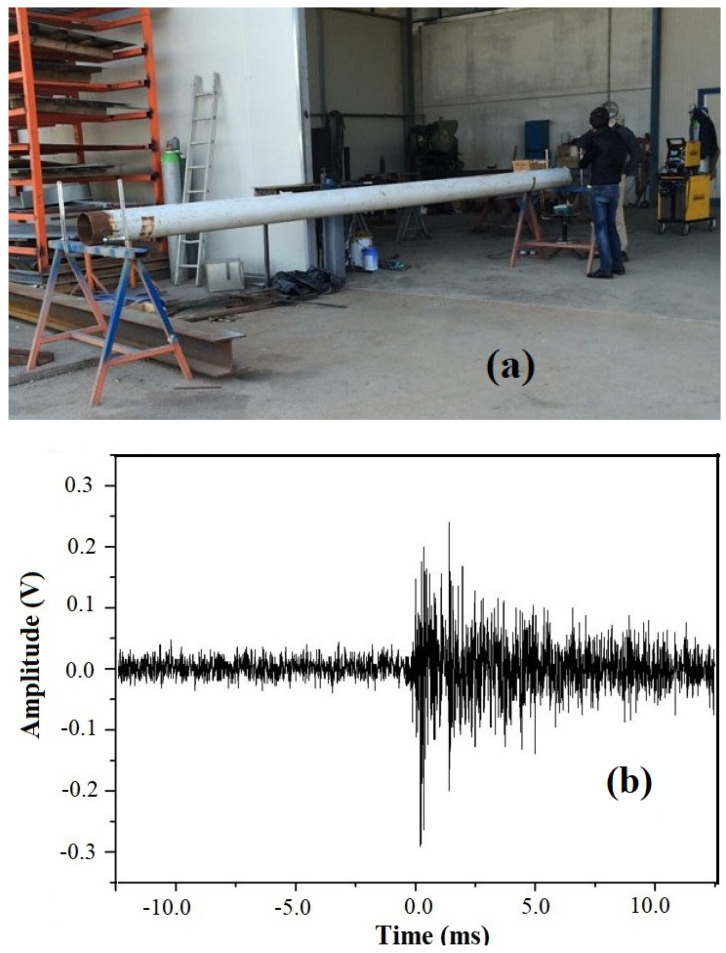
(**a**) Photo of the hollow pipe on which we first tested our sensor assembly. The pipe, white in color, was held by two supports at its ends. The pipe length was 5.5 m, its diameter was 0.3 m, and its thickness was 0.01 m; (**b**) sensor response: negative values of the horizontal scale refer to the time before the sensor responded to the excitation, which is zero on the scale.

Let us discuss now the result of the first attempt that we made to detect the excitations induced in a steel pipe. In Figure 2a, we see a 5.5 m-long, 30 cm in diameter, 1 cm-thick pipe (white in the photo) laying on supports placed close to its ends. We fixed our sensor at one end of the pipe by the magnets while, close to the other end, we applied excitations just by letting a piece of metal weighing 10 g fall on the pipe from a height of 2 cm. In these conditions, the strength of the percussive excitation was 0.1 N and its potential energy was 1.96 mJ. The response of our sensor from the other end of the pipe is shown in Figure 2b, where one can see that it generates a burst of voltage pulses having a maximum amplitude modulus of 300 mV.

A signal amplitude on the scale of hundreds of millivolts, such as the one we recorded, which was obtained for such a tiny excitation, is promising for detecting strong percussive effects generated kilometers away from the sensor. For this purpose, however, a prompt transmission from the piezo sensor (or from arrays of these) shall be important, and one possibility to do so is to transmit information to remote sensors through ether. In the next section, we will see how we solved the problem of transmitting the information remotely.

## 3. Shaping Sensors Output Signal and Remote Sensing

**Figure 3:**
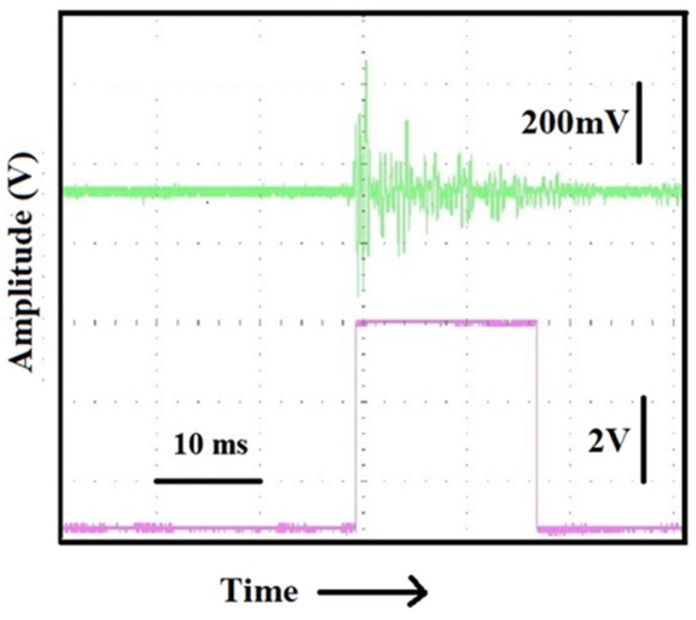
Traces of the signal detected on a multichannel scope by our piezo sensor before (upper, green trace) and after the treatment transformed it into an 18 ms pulse ready to be conveyed to a transmitter (lower, violet trace). Vertical scales are 200 mV/div for the upper trace and 2 V/div for the lower trace.

Let us see now how it is possible to shape a signal such as the one shown in Figure 2b to transform it into a rectangular pulse to be handled and eventually transmitted at a distance. When a vibration is detected by the piezoelectric, the signal it generates is integrated by a charge amplifier (shaper) and then discriminated in amplitude and sent to a monostable multivibrator (one-shot) to obtain a logic signal (trigger) with a fixed duration, as shown in Figure 3, where we see that in the upper trace, the signal originated from the piezoelectric, and in the lower trace, the “treated” signal transformed into a trigger pulse.

**Figure 4:**
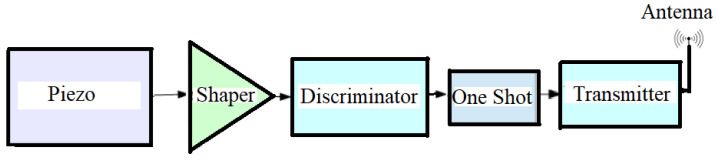
Flow chart of the system we have set up in order to transmit in the ether the signal generated by the piezoelectric sensor.

In Figure 4, a block scheme describes how the pulse of Figure 3 (upper trace) can be shaped and transmitted to a remote receiver. The operation of the set-up is as follows: in the normal state, the transmitter is in the standby state and requires a very low current for its operation. When a vibration/excitation is detected, the piezo generates a signal that is integrated by the charge amplifier (shaper), discriminated in amplitude, and sent to a monostable vibrator (one-shot) to obtain a logic signal (trigger) of predetermined duration, as shown in Figure 3 (bottom). This signal is sent to one of the GPIO pins of a transmitter board, which, receiving the signal, switches from the idle state (idle) to the transmission state (wake). The transmitted alarm signal, traveling on a high-frequency carrier, is capable of traveling tens of kilometers in the ether and can consist of sending any string of characters preset during the programming of the board itself.

**Figure 5:**
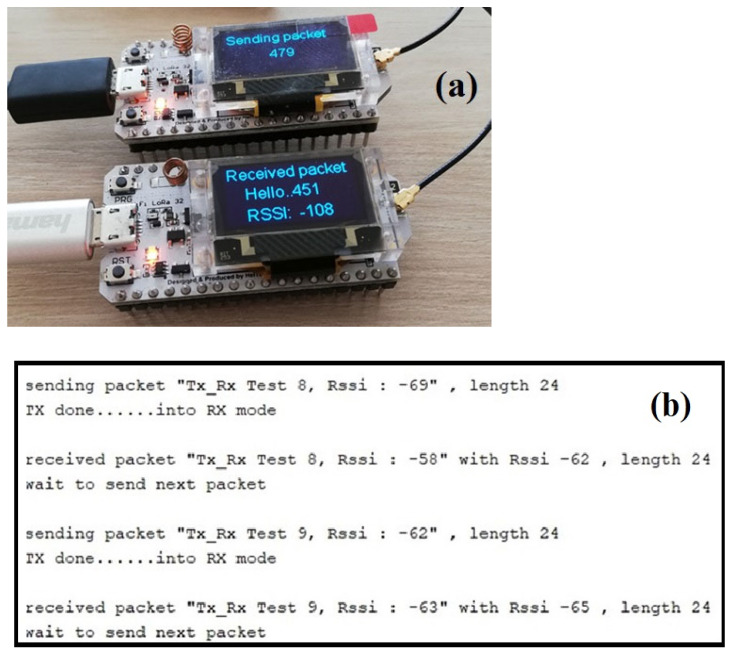
In the upper panel, (**a**) we see the two transmitter and receiver boards in the transreceiver configuration; one is set in the “Sending Packet” mode, the other in the “Receiving Packet” mode. The lower panel (**b**) shows typical computer displays tracing the activity of the boards.

The block diagram of Figure 4 was realized using a high-impedance charge amplifier LMC6041 (Texas Instruments, Dallas, TX, USA) (the shaper), collecting the signal generated by the piezo and sending it to the fast discriminator LM311 (Texas Instruments), which, in the presence of a signal, above a preset threshold, causes a monostable (one-shot) multivibrator, to generate a logic pulse of a preset width from an RC network, which represents the trigger signal. The one-shot is realized with two NOR gates of IC 74HC02 along with a capacitor and resistor determining the width of the pulse. The transmitter and receiver consist of a Heltec ESP32 LoRa V3 board (Heltec Automation, Chengdu, China), an advanced development module designed for IoT applications that integrates WiFi, Bluetooth, and LoRa (Long Range) transmission connectivity in a very small size (50.2 mm × 25.5 mm × 10.2 mm). Based on the ESP32-S3FN8 dual-core Xtensa LX7 32-bit 240 MHz microcontroller (Heltec Automation, Chengdu, China) it offers a powerful combination of performance and functionality. Transmitter and receiver board are shown in Figure 5.

The Semtech SX1276 LoRa chip-based integrated RF transmitter/receiver module (Heltec Automation, Chengdu, China) supports long-range, low-power (+22 dBm) communication [31,32,33]. The allowed radio transmission frequencies vary for different regions: 433 MHz (Asia), 868 MHz (Europe), 915 MHz (North America). Receiving sensitivity is −148 dBm. Both boards, as we see in Figure 5, are identical in size and features and can be programmed through a USB interface in a transmitter, receiver, or transreceiver configuration. The boards can interface with microcontrollers through UART, SPI, or 12C communication ports. The receiver module can recognize which of the piezos placed on pipe-lines/bars has been stressed and thus which section of the elements has been affected by an event. This information is sent to a computer in real time. In receiver configuration the board gets the signals transmitted by the devices configured in transmission, decodes the data, and sends it to a microcontroller or computer for further processing and eventual on-screen display of both transmission parameters and data. In the lower panel of Figure 5 in the first line Tx_Rx Test 8 indicate the test number for transmission (Tx) and receipt (Rx); the amplitude of the signals is recorded (−69 dBm) and also the time-length of the packet (24 byte). Our “transmitting-receiving” protocol was checked to a distance of 300 m, but the same procedure was tested over hundreds of kilometers on space ballon atmospheric research; claims exist that the protocol has been tested at distances above of 1300 km [34]: this is not a scientific publication, and surely needs to be confirmed, but records in this competitive field are transmitted using the fastest media available for communication.

## 4. Characterization of Excitations on Pipes, Bars, and Ropes

**Figure 6:**
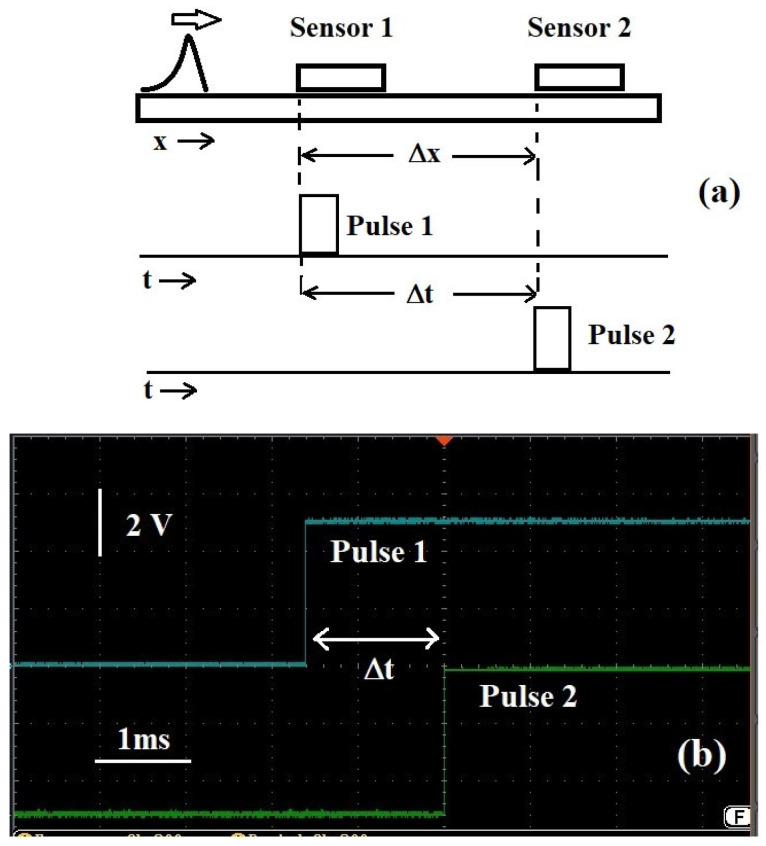
(**a**) Sketch of the protocol for measuring the time it takes for an acoustic excitation to cover the space between the two sensors; (**b**) bare oscilloscope display of the consequence of the process.

Using the rectangle-shaped signals shown in Section 3, the propagation of the acoustic excitations along pipes, bars, and ropes is characterized and the velocity of sound in the three cases can now be extracted. Two sensor assemblies are positioned at different distances on the metallic structures and a light “pulse” of acoustic excitation is generated close to the ends of the structures, as shown in Figure 6.

The output pulses of the two sensors feed two channels of the multi-trace oscilloscope RSPRO IDS1104B, 100 MHz (RS Components, Milano, Italy) on which we read and acquire the two signal traces. The time taken for the excitations (idealized as a wave packet with an arrow on it in the figure) to travel between the two sensors is measured from the time interval between the beginning of the pulses generated by the sensors, as sketched in Figure 6a. In practice, however, the time lengths of the pulses are much longer than the delay between them. A typical oscilloscope display from which one determines the time delays is shown in Figure 6b, where one can see just the rise of the two pulses and the time delay between them.

**Figure 7:**
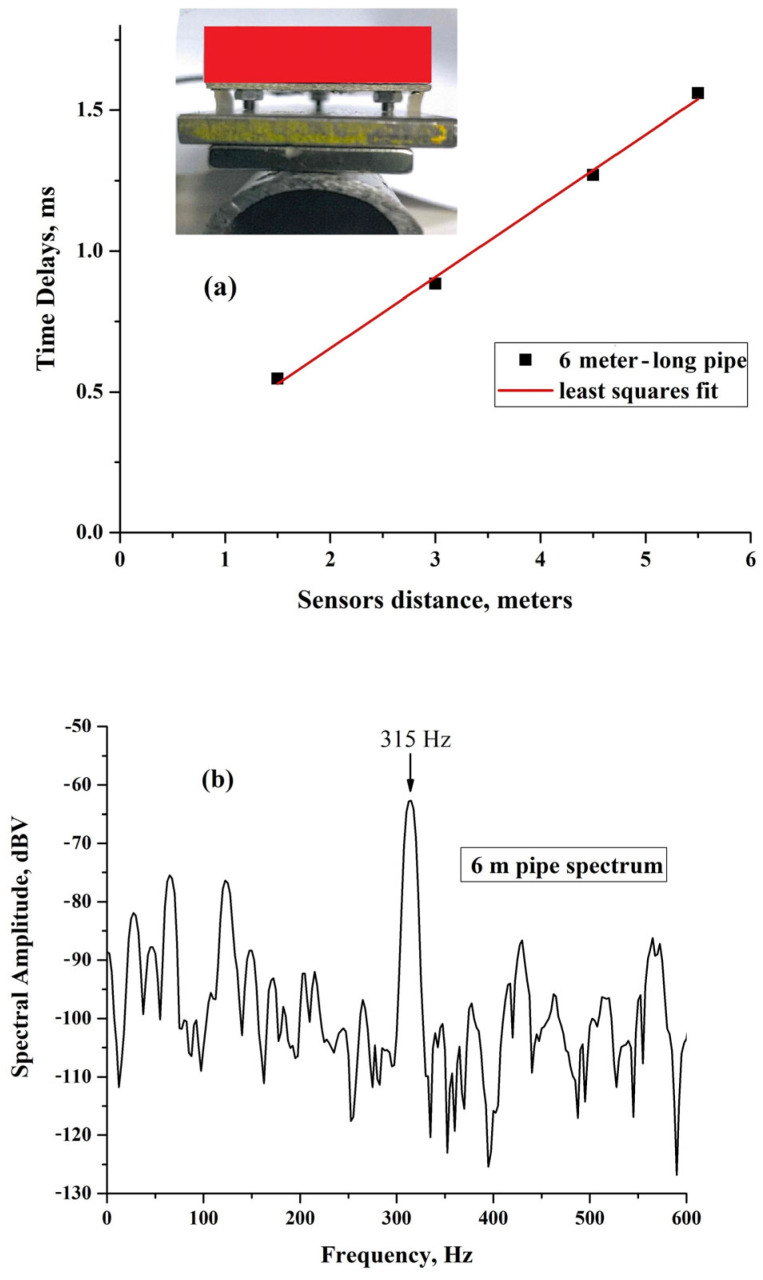
(**a**) Dependence of the time delays between the pulses generated by two sensor assemblies on the 6 meter-long pipe. The inset shows a photo of the cross-section of sensor positioning with the long side of the transmitting plate parallel to the pipe; (**b**) low-frequency spectrum showing the component corresponding to the fundamental frequency of the vibrating string model.

The measurements described in the previous paragraph were performed on a 6 m-long iron pipe with a diameter of 3.3 cm and a thickness of 0.3 cm, as shown in Figure 7a. These results, like those for the bars and ropes that will follow, were obtained by positioning the sensors as shown in the inset of the figure, with the long direction of the transmission plate parallel to the pipe. For the pipe, the sensors were positioned at relative distances, which were, respectively, 1.5 m, 3 m, 4.5 m, and 5.5 m. 

The number of measurements, 10 at least, was increased when it was considered necessary to have more statistics. In spite of this, however, the statistical errors associated with the measurements are of the order of the symbol of the data in the plot. One can see in Figure 7a that a linear relation between space and time delays exists and the least square fit of the data returns a velocity v_P_ = (3954 ± 3.6%) m/s.

The velocity v_P_ corresponds to acoustic excitations traveling along the pipe and was checked by determining the spectrum of the pipe. As for the pulse delay measurement, the spectrum was obtained by applying a slight percussive excitation at the end of the pipe, but only one sensor was necessary in this case. In the portion of the low-frequency spectrum, shown in Figure 7b, one can see a component rising 45 dBV above the noise level at 315 Hz. This frequency, considering L = 6 m, can be identified as the fundamental frequency of the vibrating string pattern, namely f = v_P_/2 L = 329 Hz; the two values (315 and 329 Hz) are consistent within the fitting error on v_P_ and the width of the spectral line. This frequency can even be seen as the first harmonic of the Fourier spectrum of a pulse traveling back and forth along a finite length L.

**Figure 8:**
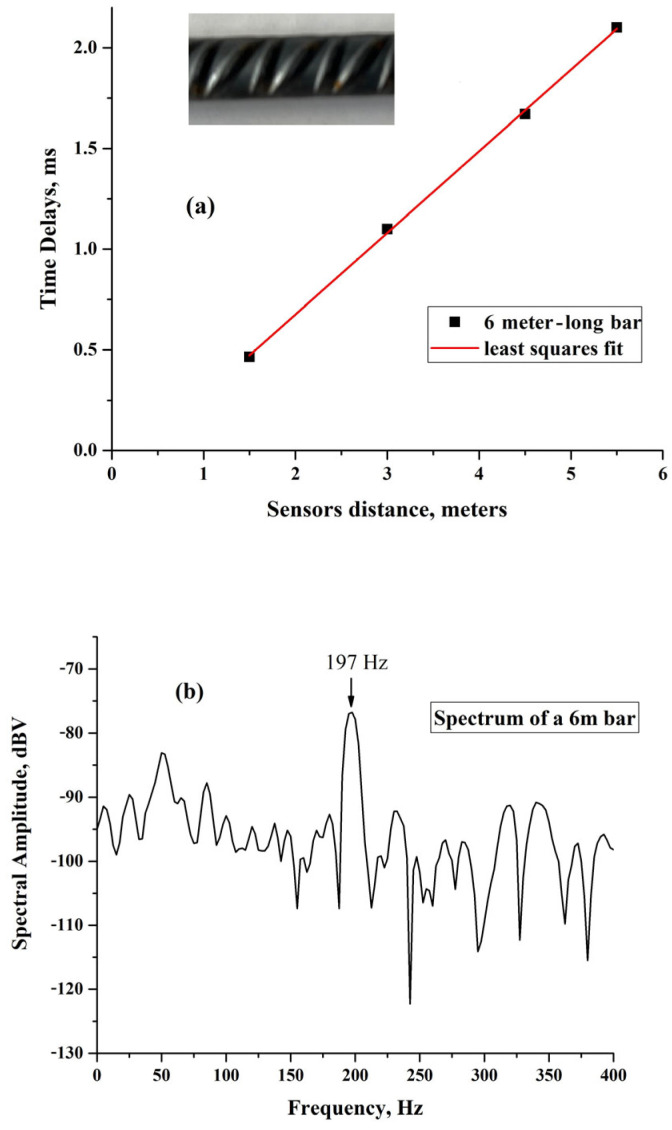
(**a**) Time delays between sensor signals placed at different distances on an iron bar. The inset shows the bar surface. (**b**) Low-frequency spectrum (portion) of the excitations of the bar.

Figure 8a shows the results obtained by placing the piezo sensors at distances of 1.5 m, 3 m, 4.5 m, and 5.5 m on a 6 m-long, 0.8 cm in diameter iron bar, of the type embedded in concrete in constructions. Even in this case, a linear dependence of the time delays on the distance between the sensors is obtained and from the least square fit to the data, and the slope it returns for the straight line, a propagation velocity v_B_ = (2470 ± 1.7%) m/s is extracted.

In Figure 8b, we see that the low-frequency spectrum of the bar exhibits a spectral line at 197 Hz, which is consistent, considering the errors in the velocity and the width of the spectral line, with the value expected for the fundamental mode of the free end resonance of the bar obtained from the above-extrapolated velocity and length of the bar, namely f = v_B_/2 L = 206 Hz.

**Figure 9:**
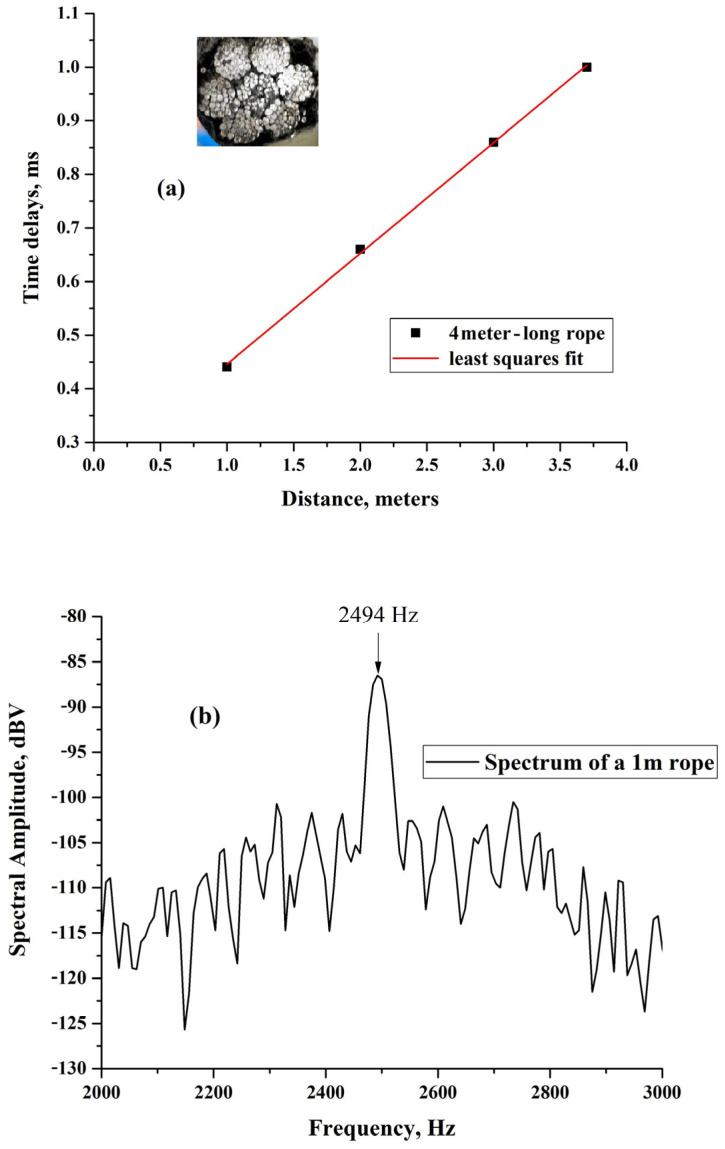
(**a**) The time delays between the 4 m-long rope. The inset shows a section of the seven wire rope. (**b**) The spectrum of a 1 m-long rope, consistent with the propagation velocity determined from the time delay measurements.

The results obtained on a 4 m-long, 2 cm-diameter, seven-wire-galvanized steel rope purchased from MITARI Hijstechniek (Eindhoven, The Netherlands) are shown in Figure 9. The results were obtained by positioning two sensors at distances of 1 m, 2 m, 3 m, and 3.7 m. When care is taken to apply excitations of the same power, the results for the time delays are reproducible. We can see that even here, the results represent a linear dependence expected like in the previous cases, indicating a reliable relation between time and space.

The line between the points is again the least square fit to the data, and from its slope, we extract that the velocity of acoustic excitations in the rope is v_R_ = (4830 ± 1.6%) m. Unfortunately, for the four-meter-long rope, it was not possible to observe the resonance in the spectrum as in the two previous cases. However, we did observe it on a one-meter-long rope of the same batch and quality, and the result is shown in Figure 9b. We can see in the figure a spectral line at 2494 Hz within 3%, consistent with the frequency of the fundamental mode f = v_R_/2 L, expected from the above-determined v_R_, and L = 1 m; here again, we see the fundamental frequency of the excitation spectrum of the vibrating string model for a 1 m-long rope.

The results of this section indicate that the detection protocol based on the generation of acoustic excitations, as well as their detection through piezo sensors, is reliable and consistent with the basic physical principles and the parameters that one can expect for the propagation of the excitations themselves along the three types of metallic structures. It is worth noting that the three structures investigated (pipe, bar, and rope) are substantially different in terms of geometric and structural points of view; this demonstrates the versatility of our piezo sensor-based protocol for characterizing acoustic propagation. Specific examples will now be presented for the localization of excitations and defects along metallic structures, and more examples of the signal traces on the oscilloscope will be shown.

## 5. Locating Excitations and Defects—Examples

**Figure 10:**
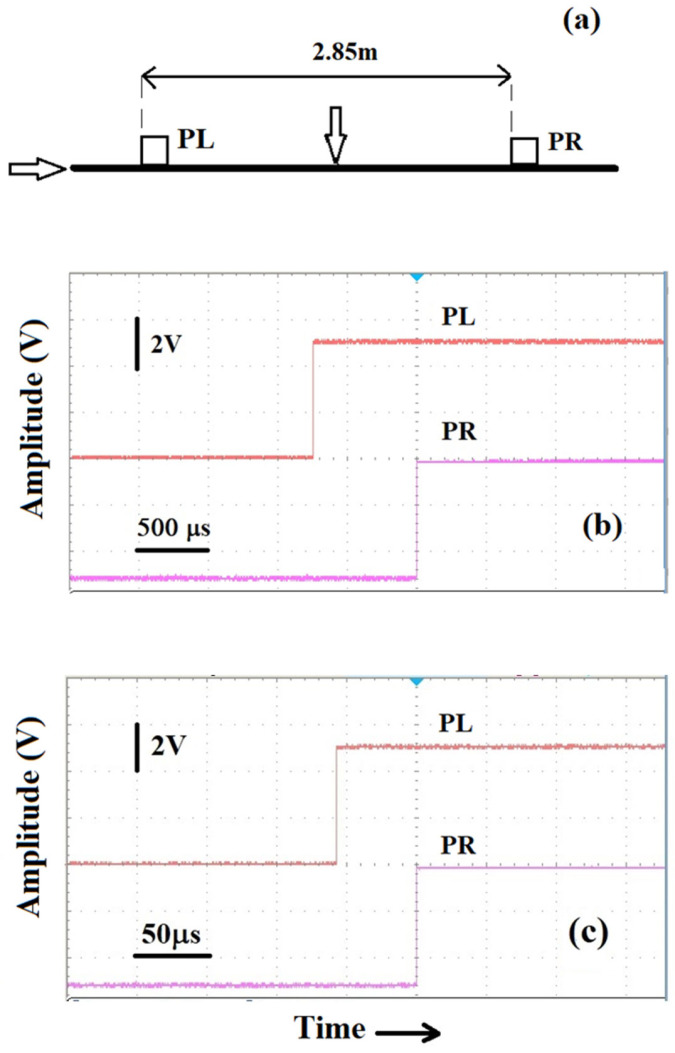
(**a**) Sketch of the experiments performed for detecting time delays between piezos. (**b**) Delay of the origin of the pulses detected by the sensors assemblies secured at a relative distance of 2.86 m, visualized on a multichannel oscilloscope, when a light percussive excitation is generated at one end. (**c**) Time delay when percussion is applied at equal distance between the piezo assemblies. Vertical scales are same for the two traces in (**b**,**c**).

For this section, we will first consider two piezo sensors sticking on the 4 m-long galvanized steel rope used in the previous section. The outputs of the two piezo sensors, discriminated to form squared impulses, feed, as before, two channels sharing the same time base of the multichannel scope. For the experiments that follow, the signals generated by the two piezos were both treated to obtain pulses like those shown in Figure 3. In the first experiment, we used a 4 m-long steel rope and the transmitting plates, along with the piezos, which were positioned 0.5 m from the ends of the rope. We note that the transmitting plates are 0.14 m long and we assume that the signals reach the sensors as soon as they excite the transmitting plate, which means that in the experimental configuration shown in Figure 10a in which we generate a light “parallel” percussive excitation on the rope, as shown by the right-pointing arrow, the effective distance between the piezos is 2.86 m. PL and PR indicate the two piezos and their position; the letters L (Left) and R (Right) represent a convenient way for us to distinguish their position on the steel rope.

The result of the light percussion is shown in Figure 10b. We see that the origin of the pulse of the piezo PR follows the one of PL after a 740 μs delay given the distance between the two sensor assemblies. Indeed, an average over several measurements in this case returned a time delay of 740 μs, which is consistent with the plot of Figure 8a. Repeating the experiment of Figure 10b but applying the “parallel” percussive excitation on the right end side of the rope, we obtained results of the same order of magnitude. However, we must point out that it is difficult to estimate precise values of propagation speed in steel ropes by relying on percussive excitations and the related transmission of the signals along the ropes, and this is evidenced in Figure 10c. In this figure, we show the time delay between the signals of the two piezos, PL and PR, measured when a light percussive excitation is applied to the rope in the center of the distance between the two sensors, as sketched by the down-pointing arrow in Figure 10a. Now, we would expect no delays between the start of the pulses since the piezos are both at 1.43 m from the excitation point but, as we see in Figure 10c, 58 μs is measured.

The reason for the time delay in Figure 10c can be attributed to the fact that for the propagation paths of the acoustic excitation in the steel ropes, one cannot expect the propagation velocities to be exactly the same in the two directions when the percussive excitation is applied in a direction orthogonal to the rope. As we shall see later in a steel bar, the same type of experiment whose results are reported in Figure 10c exhibits delays of a few microseconds.

The results of Figure 10 indicate that an experimental set-up such as the one in Figure 10a gives the possibility to discriminate where a percussive excitation has been produced. These figures clearly indicate that the time delay between the origin of the piezo pulses scales with the relative distance of the piezos from the point where the excitations were generated and that the time delay is maximum when the excitation comes from a point external to the distance between the piezos.

It is possible then that an array of piezos secured to a long bar, rope, or pipe can allow for locating the position where an excitation is applied just from the minimum of the delays measured between all sensors. In other words, if signals are coming from a linear array of equally spaced sensors on a network of pipes, for example, the shortest time delay detected from two of these sensors will locate the origin of the excitation. Since the relative error on time measurements decreases for longer distances, it is clear that this technique can increase in sensitivity and precision when the lengths of the metallic elements increase.

**Figure 11:**
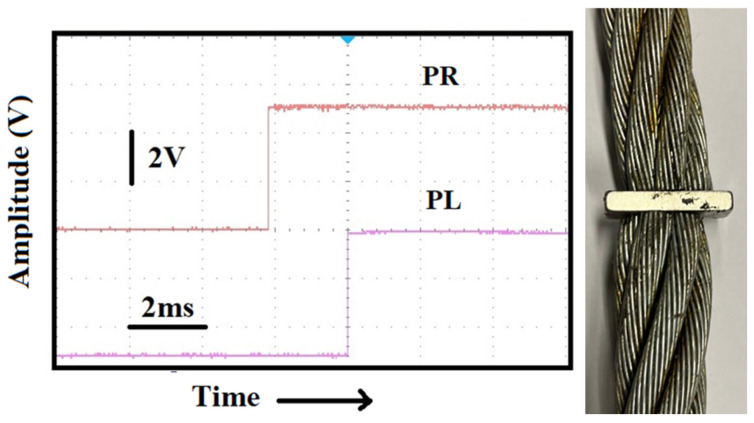
Time delay (2.1 ms) measured when the two sensors are on the sides of a defect represented by a strong magnet holding the ropes together (see photo).

Let us see now how it can be possible to locate defects along rope structures. In Figure 11, we see, in the side panel, a photo of two steel ropes (one 2 m long and another 4 m long) joined together by a strong magnet (B = 0.45 T). In the same figure, the response of the two sensors, positioned at 0.93 m from the discontinuity, is shown. In this case, an “orthogonal” percussive excitation was applied on the steel strand at half the distance (0.465 m) of the PR from the discontinuity. Now, the delay in the response of the piezo on the other side of the rope is 2.1 ms, one order of magnitude with respect to what one can obtain from a continuous rope. Even in this case, inverting the positions of the sensors, we obtained the same time delay.

It is evident from the substantially increased time delay between the origins of the pulses (2.1 ms) that the sensors reveal the irregularity situated between the ropes. The same distance between the sensors (1.4 m) on a single, uninterrupted rope would generate a time delay roughly one order of magnitude shorter, as we can tell from Figure 9a. Thus, we speculate that by measuring the time delays detected from an array of sensors placed at equal distances on bars, ropes, or pipes, one could locate irregularities in these structures just by recording anomalous time delays between the two sensors closest to the defect.

**Figure 12:**
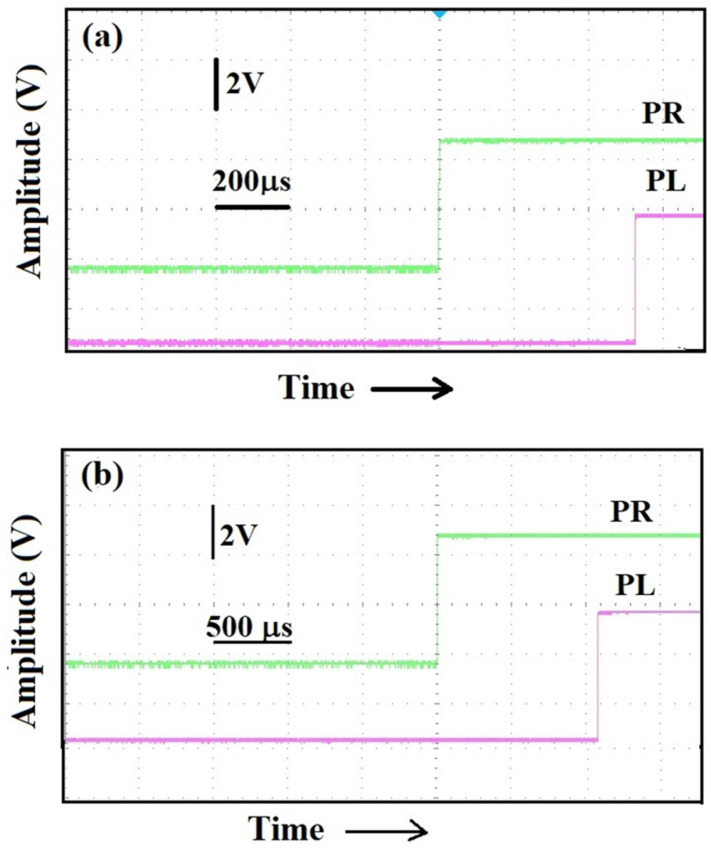
(**a**) Time delay between sensors placed 2 cm away from a 1.41 m-long steel bar as a consequence of a parallel excitation. (**b**) Time delay in the same positioning of the sensors in (**a**) when an artificial defect is introduced.

The same kind of experiments described above were performed with cylindrical steel, 0.007 m in diameter, 1.41 m-long bars. We anchored the two sensors on the steel bar at two centimeters from the ends of the bar itself and generated at one end “parallel” and “perpendicular” excitations. The result of a parallel percussive excitation generated at one end is shown in Figure 12a and we see that the delay between the pulses is 430 μs. The transmission plates were 14 cm long and were secured at 0.02 m from the ends of the bar; the effective length separating the sensors, namely the distance between the point on the bar where the excitation reached the transmission plate on the first sensor and the point where the excitation was first detected on the second sensor was 1.23 m. Given this distance, the measured time delay of 430 μs is consistent with the data presented in the plot of Figure 8a.

Generating a light, perpendicular, percussive excitation at one end of the bar, we measured the same time delay between the signals generated by the two sensors. With the bar, however, a perpendicular excitation at half the distance between the sensors always gave a few microseconds of delay, confirming that the 58 μs delay in Figure 10c is very likely due to the specific structure of the ropes.

An artificial defect, with a rough cylindrical symmetry, 2.5 cm long and 1 mm deep, was then produced in the center of the bar used for the measurement of Figure 12a. Now, the time delays between the sensor assemblies secured, as before, at 2 cm from the ends of the bar were 510 μs for “parallel” excitations at the right end of the bar, while for “perpendicular” excitations, at the same end, delays slightly above 1 ms were measured, as shown in Figure 12b. The clearly measured delays indicate the presence of an irregularity located between the two sensors since without a defect we obtained the same time delay for both perpendicular and parallel excitations, while for the parallel excitations, we obtained a 15% increase in the delay; for the perpendicular excitations, the delay increased by more than a factor of two.

## 6. Conclusions

Based on time-domain and frequency-domain measurements, it has been shown that piezo sensor assemblies can be effective in locating the positions of excitations and defects of metallic objects. Attention has been concentrated on pipes, bars, and steel ropes. Although the ropes, due to their structure, represent a “tough” candidate for testing our detection mechanism, based on the propagation speed of excitations in solids, consistent results have been obtained even for ropes. Our proposed protocol could be adequate to satisfy cases in which a non-destructive analysis of the status of bars, ropes, and pipes is necessary.

The sensitivity of our devices, their versatility, and relatively low cost (we estimate EUR 500 for each unit, including the transmitter) are such that building arrays of sensors to control rather extended networks like oil distribution pipelines can be conceived. As a tool for transferring the excitations from the investigated elements to the piezo sensors, we have employed a flat iron plate. This plate must always be flat on the side where it contacts the bottom of the three screws securing the sensor on the PCB and transmits excitations to it; however, the rest of the plate can be shaped/implemented in a way to improve the contact with specific metals geometries.

The analyses performed in Section 3, Section 4 and Section 5 show that transmission in the ether of the excitations signal (and that these start) from an array of sensors secured to bars, ropes, or pipes can enable us to determine the location of excitations and defects remotely, naturally assuming the fact that the speed of the transmission of em waves in the atmosphere is several orders of magnitude above that of sound waves in materials and, therefore, no further delays will be generated in the transmission and reception process. The time of the receiver board can be naturally synchronized to a satellite signal in a way that the detection and the analyses can be recorded in real time.

We believe that our proposal is competitive and that it is difficult to find something similar in the literature. A system of 100 piezo sensor assemblies fixed every 10 km of pipeline, for example, could help to monitor 1000 km of an oil duct with an economic load that has no match with any existing, or proposed, strategy/system. As mentioned above, the cost of each of our sensor assemblies, and signal treatments/transmissions of the piezo signal is of the order of EUR 500. Unfortunately, a “local” system capable of transmitting information in real time from large networks of elements such as pipelines conducts does not exist, and we hope it will be possible to implement a system based on the ideas herein presented.

## 7. Patents

Part of the work presented in the manuscript has been submitted to the *Ministero delle Imprese e del Made in Italy* for a utility model patent (Application nr. 202024000005079).

## Data Availability

Data presented are available, upon request, from the corresponding author.
